# Transient Osteoporosis of the Hip: Clinical and Radiological Outcomes After Combined Pharmacologic and Biophysical Therapy

**DOI:** 10.3390/jcm14217879

**Published:** 2025-11-06

**Authors:** Calogero Puma Pagliarello, Vito Pavone, Antonio Kory, Luciano Costarella, Antonio Buscema, Gianluca Testa, Corrado Ciatti

**Affiliations:** 1 Department of Orthopedics and Traumatology, Guglielmo da Saliceto Hospital, Via Taverna 49, 29121 Piacenza, Italy; c.pumapagliarello@ausl.pc.it; 2 Department of General Surgery and Medical-Surgical Specialties, A.O.U. Policlinico Rodolico-San Marco, University of Catania, Via Santa Sofia 78, 95123 Catania, Italy; vito.pavone@unict.it (V.P.); antonio.kory@gmail.com (A.K.); lcostarella@yahoo.it (L.C.); antonio.buscema@hotmail.it (A.B.); gianpavel@hotmail.com (G.T.); 3 Department of Medicine and Surgery, University of Parma, 43126 Parma, Italy

**Keywords:** bisphosphonates, electromagnetic field, hip transient osteoporosis, Neridronate, pain measurement, treatment, vitamin D

## Abstract

**Introduction**: Transient osteoporosis of the hip (TOH) is a rare, self-limiting disorder characterized by acute hip pain and reversible osteopenia. The aim of this study was to evaluate clinical outcomes following treatment with Neridronate, Clodronic Acid, Cholecalciferol, and pulsed electromagnetic field therapy (PEMF). **Materials and Methods**: A total of 45 patients presenting with non-traumatic hip pain were screened using a standardized diagnostic protocol. Magnetic resonance imaging (MRI) identified 8 patients (17.8%) with transient osteoporosis of the hip (TOH), who were subsequently enrolled in this analysis. Pain was evaluated using the Visual Analog Scale (VAS). Patients received a three-phase therapeutic protocol, including pharmacological therapy and PEMF. Clinical evaluations using the Harris Hip Score (HHS) were performed monthly, and follow-up MRI was conducted at the end of treatment. **Results**: We identified 8 cases of TOH (17.8%); the mean baseline HHS for these patients was 68.5 (range 51–83, SD 10.36). Pain reduction became evident within the first month of treatment. At the end of treatment, clinical improvement was observed in 7 patients, with mean HHS increasing to 88.0 (range 67–95, SD 8.84). Post-treatment MRI demonstrated complete resolution of bone marrow edema in all patients. One patient developed avascular necrosis despite therapy and required surgical intervention. **Conclusions**: TOH remains a controversial condition in terms of diagnosis and treatment. Early diagnosis and timely intervention are essential to progression to osteonecrosis. A combined therapeutic approach using bisphosphonates, vitamin D, and PEMF appears effective in reducing symptoms, promoting bone healing, and ensuring good patient compliance.

## 1. Introduction

Transient osteoporosis (TO), also called transient bone marrow edema syndrome, is a rare, self-limiting syndrome characterized by joint pain and osteopenia [1]. It mainly involves the proximal epiphysis of the femur, but less frequently it may also affect the knee, ankle, and foot [2,3]. Transient hip osteoporosis (TOH) usually occurs in patients between the ages of 30 and 50, and men are three times more affected than women [4]. Women tend to be more affected during the third trimester of pregnancy and in the postpartum period [4]. In recent years, the understanding of the pathophysiological mechanisms underlying transient osteoporosis of the hip (TOH) has significantly evolved [1,5,6,7]. Several hypotheses have been proposed, ranging from microvascular dysfunction and bone marrow ischemia to transient regional osteoporosis triggered by mechanical or hormonal factors. Despite these advances, the precise etiology remains elusive, which explains the variability in treatment approaches reported in the literature.

Although TOH affects a younger demographic, it arises within the broader context of skeletal fragility. Osteoporosis imposes a substantial burden on older adults, with hip fractures driving excess mortality, disability, and healthcare costs worldwide [8]. Recent modeling work has underscored the projected rise in hip fracture incidence with population aging, reinforcing the clinical priority of timely recognition and prevention across the life course. These epidemiologic trends contextualize the need for accurate diagnosis of hip pain etiologies, including TOH versus early osteonecrosis, to avoid delayed care and downstream morbidity.

TOH is supposed to have three stages. The first stage has an acute onset of hip pain due to edema potentially induced by trauma, neurovascular dysfunction, transient hyperemia, or microfractures. The second stage is characterized by increased resorption and demineralization of the bone. The final stage consists of clinical and radiographic resolution of the process [7].

It is not certain whether TOH is an isolated disease or represents the initial stage of avascular necrosis [9]. MRI is the best method to detect bone marrow edema; it is sensitive enough to detect TOH 48 h after symptoms begin. Characteristic MRI findings of TOH include delayed peak enhancement of edematous marrow, a homogeneous pattern of enhancement with no clear border, a diffuse pattern of edema with no focal defect, the presence of an irregular band of low signal intensity due to stress fracture, and the lack of subchondral changes on T2-weighted or contrast-enhanced T1-weighted images. The typical location of the edema is in the femoral head and may also involve the femoral neck and intertrochanteric region, often associated with joint effusion. The absence of subchondral changes is an indicator of TOH. In all three phases of TOH, due to increased blood flow and capillary permeability, an augmentation in radionuclide uptake can be observed, persisting for weeks after clinical improvement [7].

At present, there is no single treatment protocol for TOH, and the therapeutic schemes proposed in the literature are multiple. Medical treatments include bisphosphonates, calcitonin, teriparatide [10,11,12,13,14], and pulsed electromagnetic fields [15,16].

Moreover, the overlap between TOH and early-stage avascular necrosis of the femoral head continues to pose diagnostic challenges. This overlap often delays targeted treatment and highlights the importance of combining clinical, radiological, and functional assessments for accurate diagnosis and monitoring.

The aim of this study was to evaluate the clinical and radiological outcomes following treatment with Neridronate, Clodronic acid, Cholecalciferol, and Pulsed Electromagnetic Fields.

## 2. Materials and Methods

### 2.1. Study Design and Population

This retrospective observational study included 45 consecutive patients who presented with non-traumatic hip pain and were evaluated according to our institutional diagnostic and therapeutic protocol. The study population represented a heterogeneous group in terms of physical activity and occupational background, reflecting the typical demographic distribution of transient osteoporosis of the hip (TOH) in routine orthopedic practice. All patients underwent standardized clinical, radiographic, and magnetic resonance imaging (MRI) evaluation.

### 2.2. Inclusion and Exclusion Criteria

Inclusion criteria were (1) age between 18 and 55 years; (2) non-traumatic onset of hip or groin pain lasting ≥ 2 weeks; (3) normal or nonspecific radiographic findings without subchondral collapse; (4) MRI examination within two weeks of presentation.

Exclusion criteria were (1) previous hip fracture or surgery; (2) inflammatory, infectious, or neoplastic diseases; (3) chronic corticosteroid therapy or metabolic bone disease other than vitamin D insufficiency; (4) MRI findings consistent with avascular necrosis (AVN) or stress fracture. Pregnancy and postpartum status were recorded but not excluded a priori.

### 2.3. Imaging Protocol and MRI Diagnostic Criteria

MRI was performed using T1-, T2-, and STIR-weighted sequences in all patients. The diagnosis of TOH required diffuse bone marrow edema of the femoral head, often extending to the neck or intertrochanteric region, characterized by low T1 and high T2/STIR signal intensity, a homogeneous or patchy pattern without a distinct subchondral serpiginous band, absence of subchondral collapse, and preservation of articular cartilage.

Early AVN was excluded if any of the following features were present: a well-demarcated subchondral low-signal crescent on T1 (“double-line sign”), focal subchondral defect, or localized perfusion abnormality on contrast-enhanced images.

### 2.4. Therapeutic Protocol and Rationale

Eight patients were diagnosed with TOH and treated according to a standardized three-phase protocol integrating pharmacological therapy, vitamin D supplementation, pulsed electromagnetic field therapy (PEMF), and progressive rehabilitation.

Phase 1 (Month 1): Patients observed complete non-weight bearing and performed isometric exercises to preserve muscle trophism. PEMF was applied for 12 h daily. Pharmacologic therapy included two intramuscular cycles of Neridronate 25 mg for four consecutive days, repeated after a 7-day interval. Cholecalciferol 25,000 IU was administered once monthly.

Phase 2 (Months 2–3): Partial weight bearing (30% body weight) was allowed. Active and passive mobilization exercises were introduced. PEMF was continued for 10 h daily, and Clodronic acid 200 mg intramuscularly was administered twice weekly for 8 weeks, combined with monthly vitamin D supplementation.

Phase 3 (Month 4): Full weight bearing and non-competitive sports activities were progressively resumed. Rehabilitation focused on proprioceptive retraining, muscle strengthening, and gait correction.

The therapeutic sequence was based on pharmacodynamic rationale: Neridronate was used initially for its rapid antiresorptive and analgesic effect, followed by Clodronic acid to maintain bone remodeling inhibition with a safer and more cost-effective profile for long-term outpatient use. The higher initial PEMF exposure (12 h/day) aimed to maximize early anti-edematous and osteoanabolic effects, progressively reduced to 10 h/day as mechanical loading increased.

The chosen PEMF regimen (12 h/day in phase 1, 10 h/day in phase 2) was based on previous experimental and clinical reports indicating that prolonged low-frequency exposure enhances osteoblastic activity and accelerates resolution of bone marrow edema [15,16]. Comparable daily exposure durations have been reported in osteonecrosis and bone marrow edema syndrome protocols.

### 2.5. Outcome Measures and Follow-Up

Pain intensity was evaluated with the Visual Analog Scale (VAS), and hip function was assessed using the Harris Hip Score (HHS) at baseline and at monthly intervals (Table 1). MRI follow-up was performed at the end of treatment to confirm resolution of bone marrow edema or identify progression to AVN. Baseline MRI was performed at diagnosis and a second MRI was obtained at the end of the 4-month therapeutic program to document edema resolution or progression.

Given the small sample size (n = 8), all statistical comparisons were interpreted with caution and considered exploratory rather than confirmatory. The analyses were intended to illustrate trends rather than infer population-level effects.

### 2.6. AI Statement

Artificial Intelligence Statement: The authors used OpenAI’s ChatGPT (GPT-5, OpenAI, San Francisco, CA, USA) to assist in improving the English grammar, style, and clarity of the manuscript. The final scientific content, data interpretation, and conclusions were entirely the responsibility of the authors.

### 2.7. Statistical Analysis

All data were analyzed using IBM SPSS Statistics software, version 27.0 (IBM Corp., Armonk, NY, USA). Continuous variables were expressed as mean ± standard deviation (SD) and categorical variables as absolute and relative frequencies. The Shapiro–Wilk test was applied to verify the normality of data distribution.

Comparisons between pre-treatment and post-treatment clinical parameters, including pain intensity (VAS) and joint range of motion, were performed using the paired Student’s *t*-test for normally distributed data and the Wilcoxon signed-rank test for nonparametric data. Repeated measures analysis of variance (ANOVA) was used to evaluate longitudinal variations in pain and functional recovery across the three treatment phases.

Categorical variables, such as the presence or absence of muscle hypotrophy and radiographic alterations, were analyzed using the chi-square test or Fisher’s exact test when appropriate. A two-tailed *p*-value < 0.05 was considered statistically significant.

Missing data were handled using pairwise deletion to preserve the maximum number of observations for each analysis without introducing bias.

## 3. Results

Radiographic examination revealed 15 cases of osteoarthritis (33.3%), 8 cases of femoroacetabular conflict (17.8%), and 5 cases of avascular necrosis of the hip (11.11%). Pathological radiographic changes were not observed in the remaining 17 patients (37.8%). An MRI examination, performed as a second-level investigation, revealed 3 cases of trochanteric bursitis (17.6%, 6.7% of total cases), 6 cases of adductor tendinopathy (35.2%, 13.3% of total cases), and 8 cases of THO (47.05%, 17.8% of total cases). These 8 patients were recruited for our study. The temporal evolution of symptoms generally mirrored the imaging findings. Patients typically reported a reduction in pain intensity during the second month of therapy, which corresponded with decreased bone marrow edema on MRI and progressive improvement in joint mobility.

The mean age of these 8 patients was 43.88 years (range 31–52), with 5 men (62.5%) and 3 women (37.5%). Two patients showed bilateral involvement (25%). Hypotrophy of the quadriceps and gluteal muscles was observed at the first clinical check-up in patients with unilateral transient edema, with an average reduction in thigh circumference of −1.73 cm (SD 1.52) compared to the healthy limb.

The average HHS was 68.5 (range 51–83, SD 10.36) (Table 2). MRI showed reduced signal intensity in T1-weighted images and an area of increased intensity in T2. No reduction in joint space or subchondral erosions were observed. During treatment, a progressive reduction in pain symptoms was reported starting from month 1 (Table 3).

Pain intensity, measured by the Visual Analog Scale (VAS), showed a progressive and consistent reduction across all patients except one. At the cohort level (n = 8), VAS decreased from 6.88 ± 1.55 at baseline to 4.50 ± 1.93 at 1 month, 3.12 ± 2.59 at 2 months, and 1.38 ± 2.77 at 3 months, indicating progressive pain relief in parallel with functional gains (Table 3). Clinical improvement was observed in 7 cases, with relief of painful symptoms and an increase in HHSs (average value 88.00, range 67–95, SD 8.84) (Table 1). However, patient n.8 experienced clinical worsening, with reduction in the HHS value and persistence of pain. This last patient (male, 52 years) presented with baseline HHS 71 and VAS 8, with MRI consistent with diffuse bone marrow edema and no subchondral band. Despite adherence to the protocol and scheduled PEMF exposure, VAS remained 8–9 over the first 3 months and HHS declined to 67. Follow-up MRI revealed a new subchondral low-signal band consistent with early osteonecrosis, and the patient underwent core decompression with PRP. No deviations from dosing, weight-bearing instructions, or rehabilitation sessions were documented.

Functional recovery was evaluated not only in terms of pain relief but also by observing patients’ ability to resume normal daily activities. By the end of treatment, most patients reported improved autonomy in walking, stair climbing, and transitional movements, confirming the overall efficacy of the therapeutic protocol.

The mean pre-treatment Harris Hip Score (HHS) was 68.5 ± 10.36, and the mean post-treatment HHS increased to 88.0 ± 8.84. The values obtained at HHS before and after treatment were compared using the Wilcoxon signed-rank test, which showed a statistically significant increase in HHS values after treatment (*p* = 0.017). The MRI performed at the end of treatment showed complete resolution of edema in all patients. However, in patient n.8, the appearance of a necrotic area in the femoral head was noticed. Avascular necrosis of the head was therefore diagnosed, and the patient was subsequently treated with core decompression and augmentation with PRP.

Statistical analysis confirmed a consistent trend of clinical improvement across all measured parameters. The reduction in VAS scores over time was paralleled by progressive gains in hip flexion and abduction, reflecting both analgesic and biomechanical recovery.

## 4. Discussion

TOH research typically involves small, heterogeneous cohorts, as the condition is frequently underrecognized and inconsistently managed across medical specialties. The condition was first described by Curtiss and Kincaid in 1959 as transient osteoporosis [4,17,18]. In 1968, Lequesne reported the disease in French literature under the term “algodystrophy” [4]. In 1988, Wilson proposed the term “medullary edema” based on specific pathological MRI findings. Authors refer to TOH using various terms, such as “Transient osteoporosis,” “Algodystrophy,” “Bone marrow edema,” and “Avascular necrosis.”

The clinical course observed in this study aligns with the self-limiting nature of TOH described in previous literature. However, the adoption of a structured therapeutic protocol combining pharmacological therapy, protected weight bearing, and progressive rehabilitation appears to shorten recovery time and reduce symptom recurrence.

The term transient osteoporosis usually derives from radiographic evaluations, which become visible only 3–6 weeks after symptom onset. Today, early diagnosis is possible thanks to MRI [19]. Algodystrophy, now identified as complex regional pain syndrome (CRPS), affects the distal extremities and is characterized by erythema, functional impairments, edema, and vasomotor and sweating changes [20].

TOH is a self-limiting condition; therefore, clinical symptoms should guide the surgeon in the therapeutic approach. Currently, therapeutic strategies include mild analgesics, NSAIDs, and partial weight bearing. Some authors believe that glucocorticoids do not affect the remineralization process and are therefore not used routinely.

Partial weight bearing is an important part of treatment due to the loss of trabecular microarchitecture, which affects local bone strength. Therefore, weight bearing should be withheld until bone mineral density increases. However, prolonged non-weight bearing may lead to further bone loss due to demineralization [21].

Microfractures may play a role in disease onset due to the separation of bone trabeculae caused by bone marrow edema. Typically, if the cause of the edema is removed, the healing process progresses, resulting in clinical and radiological improvement. Patient compliance with conservative treatment and the use of medications to halt bone resorption or stimulate bone formation may shorten recovery time. However, if TOH does not reverse, a fracture may lead to misdiagnosis as AVN. Additionally, increased bone damage and further edema can result in bone collapse, joint changes, and necrosis [22].

It is unclear whether avascular necrosis can be considered the final stage of TO [5,8,9]. The fundamental difference between AVN and TOH lies in the lack of an active repair phase in AVN. As reported in other studies [23,24], in our study we observed a case of TO progressing to AVN, suggesting that TO may represent an early stage of AVN [5,8].

The main difference between TOH and AVN is that TOH generally resolves without consequences, whereas AVN leads to permanent joint failure due to interrupted blood supply to the femoral head [22]. The presence of soft tissue edema and joint effusion associated with TO suggests an inflammatory pathogenesis [7]. However, treatment with intra-articular or systemic corticosteroids has shown no significant effect on reducing TOH. Prednisone at 30 mg/day for four months or at doses up to 40 mg/day did not modify the natural course of the disease.

The onset of TO varies among patients and may be either gradual or sudden. A triggering event cannot usually be identified, although in approximately 10% of patients, pain occurs after unusual activity, such as a long walk [18]. Patients typically report nonspecific groin pain radiating to the thigh, aggravated by weight bearing, leading to limited walking. Clinical signs include limping due to gluteal muscle hypotrophy and functional limitations, with pain worsening during provocative maneuvers [25]. Radiographs, often used as the first diagnostic tool, may show diffuse and progressive osteopenia from the femoral head to the neck and trochanters, associated with periarticular demineralization of the acetabulum. Joint space does not appear reduced, and subchondral bone alterations are usually absent. Radiographic changes are detectable 3–6 weeks after symptom onset. In contrast, MRI can detect early bone changes as soon as 48 h after clinical onset and is the most specific diagnostic examination for TOH [26].

MRI alterations are characterized by a poorly defined area of reduced signal intensity in T1 and an area of increased intensity in T2, typically located in the femoral head but possibly extending to the femoral neck, sparing the acetabulum [27]. STIR sequences, through homogeneous fat suppression, allow better definition of the bone marrow edema area.

Early diagnosis combined with timely treatment is therefore essential. Therapeutic schemes reported in the literature are diverse and are based on the administration of bisphosphonates, teriparatide, denosumab, and iloprost.

Neridronate has proven effective in achieving rapid pain relief with a good tolerability profile [12]. The therapy then continues with Clodronate, a safer, lower-cost drug that also induces the production of anti-inflammatory cytokines [28]. Some authors have highlighted that TO may be associated with vitamin D deficiency and osteopenia [10,12,29,30]; therefore, vitamin D supplementation is indicated to prevent recurrence.

The occurrence of a single progression to avascular necrosis, despite early multimodal therapy, aligns with previous reports suggesting that transient osteoporosis of the hip may, in selected cases, represent an early reversible stage of osteonecrosis. Continuous MRI monitoring remains essential for timely identification of this transition.

The main limitation of this study is the small number of patients, which does not allow definitive conclusions regarding the efficacy and safety of the protocol, despite the clear scientific rationale supporting the proposed treatment.

Nevertheless, the variability in therapeutic approaches across studies reflects the absence of standardized guidelines for TOH management. Further multicenter trials and prospective studies are warranted to define evidence-based treatment algorithms and clarify the long-term outcomes of conservative versus pharmacologic strategies.

### Limitations of the Study

This study has several limitations that should be acknowledged. First, its retrospective design inherently limits the ability to establish causal relationships and introduces a potential risk of selection bias. The relatively small sample size and the absence of a control group may restrict the generalizability of the findings. The limited number of MRI-confirmed cases reduced the statistical power of inferential analyses, and the findings should therefore be interpreted as exploratory rather than confirmatory. Furthermore, all patients were managed within a single institutional protocol, which, although consistent, might not fully represent variations in clinical practice or rehabilitation settings across different centers. Additionally, some limitations of the present study may be related to the broader organizational and clinical challenges that emerged in the post-COVID-19 era. Reduced accessibility to rehabilitation services, delayed imaging follow-up, and increased patient reluctance to attend in-person visits might have influenced the continuity and timing of clinical evaluations. These contextual factors, although external to the study design, may have introduced minor variability in data collection and follow-up adherence [31,32].

Another limitation lies in the lack of long-term follow-up beyond the completion of the therapeutic program. Consequently, potential late recurrences or delayed complications could not be assessed. In addition, although magnetic resonance imaging was used for diagnostic confirmation, quantitative MRI parameters—such as edema volume or signal intensity ratios—were not evaluated, which could have provided more objective insights into disease progression and healing dynamics.

Despite these constraints, the study provides valuable clinical evidence by applying a standardized diagnostic and therapeutic protocol to a homogeneous patient cohort. The consistency of treatment, comprehensive follow-up, and the integration of both clinical and radiological outcomes strengthen the reliability and internal validity of the reported results.

## 5. Conclusions

TOH remains a rare and self-limiting condition whose diagnosis and management continue to pose a challenge in orthopedic practice. Although the disorder often resolves spontaneously, early recognition and appropriate therapeutic intervention are essential to minimize disability and prevent progression toward more severe complications such as femoral head collapse or avascular necrosis.

The results of this study reinforce the concept that early diagnosis, patient compliance, and close clinical monitoring are key determinants of recovery in TOH. A multimodal management strategy—integrating pharmacologic therapy, imaging follow-up, and structured physiotherapy—should be considered the cornerstone of effective treatment.

The proposed three-phase therapeutic protocol demonstrated favorable outcomes in terms of pain reduction, functional recovery, and radiological improvement, confirming the value of coordinated medical and rehabilitative approaches. While these findings support the effectiveness of conservative treatment, further prospective and multicenter studies are required to establish standardized guidelines and to clarify the long-term prognosis of this condition.

## Figures and Tables

**Table 1:** Individual patient values for Harris Hip Score (HHS) recorded at baseline and at the end of the therapeutic protocol. Individual pre- and post-treatment HHS values for the eight patients diagnosed with transient osteoporosis of the hip (TOH). Overall improvement was observed in 7 of 8 patients.

Patient	HHS Before	HHS After
1	64.0	95.0
2	71.0	89.0
3	58.0	92.0
4	83.0	90.0
5	73.0	89.0
6	51.0	88.0
7	77.0	94.0
8	71.0	67.0

**Table 2:** Descriptive statistics for the HHS values before and after treatment. Results are expressed as minimum, maximum, mean, and standard deviation (SD). The mean HHS increased significantly after treatment, indicating significant functional recovery (*p* = 0.017, Wilcoxon signed-rank test).

Variable	n	Minimum	Maximum	Mean	SD
HHS Before Treatment	8	51.0	83.0	68.5	10.36
HHS After Treatment	8	67.0	95.0	88.0	8.84

**Table 3:** VAS pain scores recorded at baseline and at 1-, 2-, and 3-month follow-up visits. Progressive reduction in pain intensity was observed across all patients, confirming the clinical efficacy of the combined pharmacologic and rehabilitative approach. A progressive decrease in VAS pain intensity was observed in 7 of 8 patients, indicating consistent clinical improvement over time. Patient #8 showed persistent pain and later developed avascular necrosis.

Patient	Baseline	1 Month	2 Months	3 Months
**1**	9	5	4	2
**2**	7	4	3	0
**3**	6	6	3	1
**4**	8	5	2	0
**5**	6	3	2	0
**6**	4	2	1	0
**7**	7	3	1	0
**8**	8	8	9	8
**Mean ± SD**	*6.88 ± 1.55*	*4.50 ± 1.93*	*3.12 ± 2.59*	*1.38 ± 2.77*

## Data Availability

The data presented in this study are available on request from the corresponding author.
